# Symmetrical peripheral gangrene after cardiac surgery: A case report and review of literature

**DOI:** 10.1097/MD.0000000000041527

**Published:** 2025-02-14

**Authors:** Xiaoliang Chen, Guofeng Shao, Shunying Zhao, Ni Li

**Affiliations:** a Department of Cardiosurgery Intensive Care Unit, Ningbo Medical Centre Lihuili Hospital, Ningbo University, Ningbo, China; b Department of Cardiothoracic Surgery, Ningbo Medical Centre Lihuili Hospital, Ningbo University, Ningbo, China.

**Keywords:** cardiac surgery, cardiogenic shock, disseminated intravascular coagulation, symmetric peripheral gangrene

## Abstract

**Rationale:**

Cardiac surgery can cause arrhythmias and low cardiac output syndrome through various mechanisms, including cardiac manipulation, systemic inflammation, myocardial hypoxia, cardioplegic arrest, and ischemia resulting from coronary or graft occlusion. Symmetric peripheral gangrene is a rare but serious complication that can occur after cardiac surgery. Here, we present a case of symmetric peripheral gangrene shortly after cardiac surgery for mitral valve replacement.

**Patient concerns:**

A 76-year-old male with rheumatic heart disease and severe mitral stenosis underwent mitral valve replacement. He experienced postoperative bleeding on the first day after the cardiac surgery. After experiencing ventricular fibrillation, cardiogenic shock, acute hepatic failure, and disseminated intravascular coagulation (DIC), he developed symmetrical peripheral gangrene on the fifth day after cardiac surgery.

**Diagnoses:**

The patient presented with postoperative bleeding, cardiogenic shock, and DIC complicated by symmetrical peripheral gangrene following cardiac surgery.

**Interventions:**

During cardiosurgery intensive care unit admission, the patient received positive inotropic agents and vasopressors, blood transfusion, and antithrombotic treatment with low -molecular-weight heparin. Because of the severe general condition of the patient, amputation was not performed.

**Outcomes:**

The patient died on the 80th day after cardiac surgery because of multiorgan failure and DIC.

**Lessons:**

Physicians should be vigilant for comorbid symmetrical peripheral gangrene in patients undergoing cardiac surgery who present with postoperative bleeding, cardiogenic shock, and DIC. Early recognition of acrocyanosis, prompt management of cardiogenic shock, correction of anemia, hemodynamic stabilization, and properly controlled use of anticoagulation may help prevent symmetrical peripheral gangrene.

## 1. Introduction

Cardiac surgery involves life-enhancing procedures in patients with severe cardiac conditions, including congenital, coronary, valvular, and structural heart disease.^[1]^ Major complications after cardiac surgery include postoperative bleeding, low cardiac output syndrome, cardiogenic shock, and perioperative myocardial infarction, which are associated with worsened clinical outcomes and mortality rates.^[2]^

Symmetrical peripheral gangrene (SPG) is symmetrical distal ischemic damage at 2 or more sites without a primary vascular occlusive disease. The syndrome is devastating and rare, and controlled studies on its etiology and management are lacking. Although it has been reported in several conditions such as sepsis, infective endocarditis, and disseminated intravascular coagulation (DIC),^[3–5]^ it is rarely associated with cardiac surgery.^[6]^

Here, we present a unique case of symmetrical peripheral gangrene following mitral valve replacement for postoperative bleeding and cardiogenic shock. We also review the literature and previously documented cases of SPG to summarize the contemporary precautions and management of this condition.

The ethics committee of Lihuili Hospital of Ningbo Medical Center approved this study. Written informed consent has been obtained from the patient’s family members to publish this case report.

## 2. Case report

A 76-year-old male (height, 155 cm; weight, 48.5 kg) had rheumatic heart disease with severe mitral stenosis, chronic heart failure, and chronic atrial fibrillation. The patient presented with symptoms of chest tightness and shortness of breath after exercise over the past 5 years, which had aggravated obviously in the past 2 months. According to the New York Heart Association (NYHA) grading standard, his cardiac function was class III. Comorbidities included hypertension, diabetes, hyperuricemia, atherosclerotic carotid plaque, and prostatic hyperplasia. There was no reported family history of peripheral vascular disease or related genetic disorders in his first- or second-degree relative. He denied a history of smoking or alcohol consumption. He denied recent travel, exposure to chemicals, or any pets. He denied any history of hematological, lung, liver, or kidney disease. On physical examination, his vital signs were as follows: temperature 36.4°C, pulse rate 50/minute, respiratory rate 14 breaths/minute, blood pressure 135/60 mm Hg, and oxygen saturation 98%. Chest auscultation revealed increased breath sounds on both sides with no dry and wet rales, an absolute irregular heart rhythm, a rumbling murmur in the diastolic period of the heart, and mild edema in both lower limbs. The remaining physical examination results were unremarkable. Transthoracic echocardiogram (TTE) showed a slightly enlarged left atrium (42 mm, NR: 19–40 mm), normal left ventricular (LV) end-diastolic diameter (40 mm), normal LV systolic function with an ejection fraction of approximately 55%, and a degree of mitral stenosis with a valve area of 0.97 cm^2^. Arterial Doppler of the upper and lower limbs was normal and did not reveal any thrombus in any of the arteries.

Mitral valve replacement was performed. The patient was weaned off cardiopulmonary bypass with dopamine 6 µg/kg/minute, epinephrine 0.06 µg/kg/minute, and norepinephrine 0.08 µg/kg/minute. Total cardiopulmonary bypass time, aortic cross-clamp time, and surgery duration were 84 minutes, 34 minutes, and 4.5 hours, respectively. Total blood loss was estimated at 500 mL, and 2.5 units of packed red blood cells were used during surgery. The patient was transferred to the cardiosurgery intensive care unit (CICU) postoperatively and was in stable condition.

On the first day of admission to the CICU, the patient presented low hemoglobin with chest drained 160 to 200 mL serohematic fluid every 2 hours to a total of 1.0 L. Laboratory tests showed hemoglobin of 57.0 g/L, platelet count of 48 × 10^9^/L, prothrombin time (PT) of 16.0 seconds, international normalized ratio (INR) of 1.29, activated partial thromboplastin time (APTT) of 47.3 seconds, fibrinogen of 2.49 g/L, D-dimer of 0.98 mg/L and lactate of 8.0 mmol/L. Due to the patient’s postoperative bleeding, packed red blood cells 3 units, fresh frozen plasma 9 units, and platelet 10 units were given.

On the fifth day of admission to the CICU, Ventricular fibrillation occurred in the patient, sinus rhythm was restored after electrical defibrillation, and hemodynamic conditions were closely monitored. The Pulse index Continuous Cardiac Output (PiCCO) monitor showed a low cardiac output (2.23 L/minute) and cardiac index (1.67 L/minute/m^2^). Laboratory data showed a platelet count of 72 × 10^9^/L, alanine aminotransferase (ALT) of 320 U/L, total bilirubin of 167.7 µmol/L, direct bilirubin of 105.7 µmol/L, prothrombin time (PT) of 54.3 seconds, international normalized ratio (INR) of 6.28, activated partial thromboplastin time (APTT) of 61.0 seconds, D-dimer of 23.93 mg/L, a lactate level of 4.3 mmol/L, and brain natriuretic peptide of 3216 ng/L. the patient developed cardiogenic shock, acute hepatic failure and DIC. Due to the hemodynamic instability, it was necessary to administer positive inotropic agents and vasopressors (dopamine 8 µg/kg/minute, epinephrine 0.08 µg/kg/minute and norepinephrine 0.32 µg/kg/minute) to maintain systolic blood pressure and mean arterial pressure goals.

**Figure 1.**
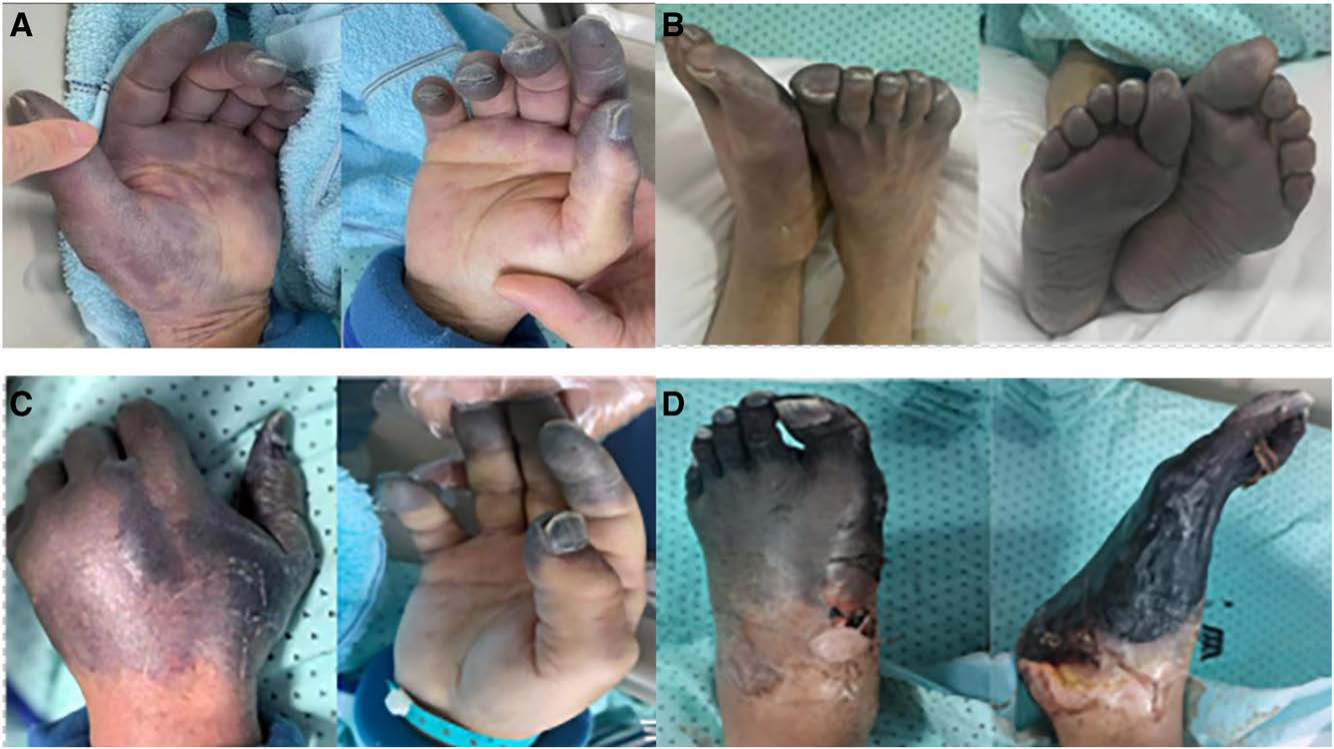
Time courses of the clinical features of symmetrical peripheral gangrene. (A, B) Extensive purple lesions and ischemic areas are seen in the acral regions of all extremities on the sixth day after cardiac surgery. (C, D) The acral regions of all 4 extremities including all the fingers and toes evolved into severely necrotic areas on the 30th day after cardiac surgery.

On the seventh day of CICU admission, grayish lesions were observed on both hands and finger pads, patchy purple lesions on both feet and toes, and cyanosis remained in the heels. The radial, ulnar, pedal, and posterior tibial pulses in the patient’s extremities were normal on Doppler examination (Fig. 1A, B). On the 31st day of CICU admission, the lesions evolved into necrotic areas at the left hand’s metacarpal and phalangeal levels and both feet’ phalangeal and metatarsal levels. On both hands, nonsuppurative, dry and well-defined necrosis was established at the distal level of the fingers. Both feet also presented with dry and well-defined necrosis affecting all toes of both feet, a superficial necrotic eschar at the left dorsal pedis level. A clinical diagnosis of SPG was made (Fig. 1C, D).

During CICU admission, the patient received positive inotropic agents and vasopressors, blood transfusion, ventilatory support, intravenous antibiotics, dialysis and antithrombotic treatment with low molecular weight heparin. Because of the severe general condition of the patient, amputation was not performed. During the delimiting of the necrotic lesion, wound care was performed with dry dressings, and limbs were covered with padded bandages. The patient with SPG had prolonged mechanical ventilation time and ICU stay time, which led to multidrug-resistant bacteria infection, and eventually died of septic shock, renal failure and DIC on the 80th day after cardiac surgery.

## 3. Discussion

Patient characteristics, surgical techniques, and postoperative complications influence cardiac surgery outcomes. The type and number of postoperative complications undoubtedly have important implications for early mortality and healthcare costs due to prolonged hospitalization. Effective and timely management of complications in the early postoperative period is essential to improving survival after cardiac surgery.^[7]^

Symmetrical peripheral gangrene is an infrequent complication of cardiac surgery. However, it is an important cause of mortality in affected patients. A mortality rate of approximately 18% to 40% has been reported, and survivors have a high frequency of multiple limb amputations.^[8]^ Symmetrical peripheral gangrene is characterized by the sudden onset of symmetrical gangrene in fingers and toes. The reason for SPG is challenging to determine, however, it has generally been divided into 2 main categories: infective and noninfective factors.^[8]^ SPG has been reported in various medical conditions such as DIC,^[9]^ septic shock,^[10]^ postoperative cardiogenic shock,^[11]^ vasopressor use, hypovolemic shock, tachycardia, acute hepatic failure,^[12]^ acute kidney injury, colloid transfusion, anemia,^[13]^ malignancy, connective tissue disorders and genetic predispositions.^[14]^ Deslivia et al^[15]^ identified diabetes mellitus, renal disease, peripheral vascular disease, and cerebrovascular disease as risk factors for SPG development. In patients with DIC and hypovolemia, the use of vasopressor drugs such as epinephrine, dopamine, and norepinephrine can cause SPG by decreasing tissue perfusion and exacerbating ischemia, leading to eventual tissue necrosis and gangrene.^[16]^

There are uncertainties about the pathophysiology of SPG. Most authors reported that SPG, a result and manifestation of DIC, has the feature of microthrombosis associated with a disturbed procoagulant-anticoagulant balance.^[17]^ The pathogenesis of SPG emphasizes the role of severely disturbed procoagulant-anticoagulant balance in patients with poor peripheral limb perfusion in the setting of hemodynamic shock. The 3 main characteristics of SPG are natural anticoagulant depletion (protein C and antithrombin), DIC, and shock (hypotension, lactic acidemia, normoblastemia, and multiple organ failure).^[14]^

There are few evidence-based recommendations for SPG treatment. However, the widely accepted objective in treating SPG is eliminating risk factors (e.g., DIC, shock, and acute or chronic liver dysfunction), preventing additional infections, removing toxins, volume resuscitation, and plasma exchange.^[14]^ A multidisciplinary management strategy is crucial, involving a team of healthcare professionals such as critical care experts, dermatologists, infectiologists, hematologists, and orthopedic surgeons. Amputation is the only definitive treatment established for gangrene in the acral areas, it should be considered after the development of a clear line of demarcation, until this moment emphasis should be placed on local wound care.^[18]^

In this case, 4 observations may have contributed to SPG. First, the patient had a brisk loss of blood which resulted in hypotension after cardiac surgery, although the blood pressure was swiftly elevated after blood transfusion and administration of vasopressors. Second, the patient presented with ventricular fibrillation and cardiogenic shock, and the administration of positive inotropic agents and vasopressors, which were widely believed to be associated with SPG, may have contributed to this. In this case, the total amounts of dopamine, epinephrine, and norepinephrine were small and far below the dangerous dose. Due to the patient’s economic reasons, the patient was not managed by mechanical circulatory support, such as extracorporeal membrane oxygenation (ECMO) or intra-aortic balloon pump (IABP). Third, acute hepatic failure and DIC occurred in the patient, which decreased natural anticoagulants, and contributed to the patient’s profoundly disturbed procoagulant-anticoagulant balance. SPG may be associated with a subsequent second episode of shock liver and DIC. Fourth, the patient had been diagnosed with diabetes for 5 years. Diabetes may be a risk factor for the development of SPG. Hence, the patient’s symmetrical peripheral gangrene was probably multifactorial rather than a single cause.

In conclusion, Physicians should be alert to the possibility of symmetrical peripheral gangrene when patients undergoing cardiac surgery present with excessive blood loss, cardiogenic shock, DIC, and administration of vasopressors is required in the perioperative period. The color of the patient’s extremities should be closely monitored, early intervention is essential if grayish lesions or purple lesions are observed on the extremities. Early intervention includes prompt management of cardiogenic shock, correction of anemia, minimizing the use of vasopressors, hemodynamic stabilization, and properly controlled use of anticoagulation. Early recognition of acrocyanosis and early intervention may be helpful in halting the progression of the pregangrenous stage to frank gangrene. Currently, there are minimal evidence-based guidelines for the treatment of SPG. Because of the rarity of SPG and its high mortality rate, multicenter, large-sample studies should be conducted to formulate proper treatment guidelines.

## Acknowledgments

This study was supported by grants from the Zhejiang Medical and Health Science and Technology Plan Project (no. 2024KY298) and the Ningbo Top Medical and Health Research Program (no. 2022030107).

## Author contributions

**Conceptualization:** Shunying Zhao.

**Formal analysis:** Xiaoliang Chen.

**Project administration:** Guofeng Shao, Shunying Zhao.

**Writing – original draft:** Xiaoliang Chen.

**Writing – review & editing:** Ni Li.
